# Spectrum-Effect Relationship-Based Strategy Combined with Molecular Docking to Explore Bioactive Flavonoids from *Sceptridium ternatum*

**DOI:** 10.3390/molecules27175698

**Published:** 2022-09-04

**Authors:** Junfeng Zhu, Haiying Ding, Like Zhong, Wenxiu Xin, Xiaojiao Yi, Luo Fang

**Affiliations:** 1 Department of Pharmacy, The Cancer Hospital of the University of Chinese Academy of Sciences (Zhejiang Cancer Hospital), Institute of Basic Medicine and Cancer (IBMC), Chinese Academy of Sciences, Hangzhou 310022, China; 2 Department of Pharmacy, Affiliated Hangzhou Xixi Hospital, Zhejiang University School of Medicine, Hangzhou 310023, China

**Keywords:** *Sceptridium ternatum*, phytochemical analysis, antioxidant activity, anti-inflammatory activity, spectrum-effect relationship, molecular modeling

## Abstract

*Sceptridium ternatum* is a herbaceous plant with significant potential for pharmaceutical and cosmetic applications. In this study, we established a spectrum-effect relationship-based strategy to investigate the bioactive basis and tissue distribution in *S. ternatum*. First, a phytochemical analysis on the ethanol extracts from roots, stems, and leaves of *S. ternatum* was performed using the colorimetric method, high-performance liquid chromatography–ultraviolet (HPLC–UV), and high-performance liquid chromatography–electrospray ionization quadrupole time-of-flight mass spectrometry (HPLC–ESI-Q-TOF-MS/MS). Then, radical scavenging assays and the lipopolysaccharide-stimulated RAW 264.7 cell model were used to estimate the antioxidant and anti-inflammatory activities, respectively. Spectrum-effect relationship analysis and molecular docking were further employed to evaluate the correlation between the phytochemical profile and anti-inflammatory activity. Our results demonstrate that *S. ternatum* leaves contained the most abundant flavonoids and exerted the best biological activities. Their IC_50_ values for scavenging 2,2ʹ-azino-bis (3-ethylbenzthiazoline-6-sulfonic acid) and 1,1-diphenyl-2-picrylhydrazyl radicals were 2.43 ± 0.13 and 5.36 ± 0.54 mg/mL, respectively. In lipopolysaccharide-stimulated RAW 264.7 cells, the leaf extract caused the greatest reduction in nitric oxide production (38.15%) and interleukin-6 release (110.86%). Spectrum-effect relationship analysis and molecular docking indicated that quercetin 3-*O*-rhamnoside-7-*O*-glucoside possessed high anti-inflammatory activity by binding with interleukin-6. In conclusion, *S. ternatum* is a rich source of bioactive flavonoids with potential for exploitation in the prevention and treatment of oxidative stress and inflammation-related pathologies.

## 1. Introduction

Flavonoids are a large group of naturally occurring polyphenolic compounds that typically contain a C6-C3-C6 skeleton structure [1]. These phytochemicals are broadly distributed in fruits, vegetables, herbs, and many other plants [2,3]. Studies have shown that the pharmacological properties of flavonoids include antioxidant, anti-inflammatory, antitumor, antiviral, neuroprotective, and cardioprotective activities [4,5,6]. In recent decades, natural products containing abundant flavonoids, such as *Matricaria chamomilla* L., have been widely utilized in the pharmaceutical and cosmetic industries because of their excellent biological benefits [7,8]. Therefore, bioactive flavonoids derived from natural sources are of particular interest.

*Sceptridium ternatum* (Thunb.) Lyon, also known as *Botrychium ternatum* (Thunb.) Sw. or *Osmunda ternata* Thunb., is a perennial herbaceous plant that is primarily distributed in eastern Asia, such as Japan, the Korean Peninsula, and China [9,10]. As a folk medicine in these areas, the whole *S. ternatum* herb is commonly used to treat asthma, cough, and fever [10]. The antiasthmatic mechanism of *S. ternatum* has been attributed to the elevated Th1/Th2 ratio and reduced cysteinyl leukotriene receptor-1 expression [11,12]. *S. ternatum* can also relieve pulmonary arterial hypertension by inhibiting NF-κB p65 and α-smooth muscle actin [13]. Additionally, *S. ternatum* has gained increasing attention because of its vast potential in the cosmetic field. For example, an external skin composition containing *S. ternatum* extract has been used for whitening, anti-aging, and moisturizing purposes [14]. In a 2,4-dinitrochlorobenzene-induced mouse model, *S. ternatum* attenuated allergic contact dermatitis-like skin lesions, which suggested that *S. ternatum* is a promising treatment option for skin inflammation [15]. Our previous study reported that *S. ternatum* is a rich source of flavonoids and shows an obvious antioxidant capacity [16]. However, studies related to the bioactive basis and underlying mechanisms of its anti-inflammatory activity are still scarce, and the tissue distribution of bioactive flavonoids in *S. ternatum* remains unknown.

Spectrum-effect relationship analysis integrates chemical profiles and pharmacological activities to identify the major active constituents in complex samples. This method has been successfully applied to reveal the material basis of traditional Chinese medicines. By correlating high-performance liquid chromatography (HPLC) chromatograms with cell inhibitory activities, Liu et al. [17] found that three compounds in *Scheflera heptaphylla* were positively correlated with the anti-hepatoma effect. Zhou et al. [18] found that ginsenoside Ro is responsible for the antiproliferative activity of *Panax ginseng*. Since *S. ternatum* contains dozens of flavonoids, and none of them have commercially available standards, spectrum-effect relationship analysis combined with high-resolution mass spectrometry identification could be a potential strategy with which to identify bioactive components in *S. ternatum*.

In the present study, we hypothesized that chemical differences in different parts of *S. ternatum* would result in different pharmacological activities. Therefore, we established a spectrum-effect relationship-based strategy to investigate the bioactive basis and tissue distribution in *S. ternatum*. First, the phytochemical profiles and biological activities of ethanol extracts from different parts of *S. ternatum* were comparatively analyzed. Then, HPLC chromatogram-effect correlation analysis combined with high-resolution mass spectrometry was performed to identify the bioactive components in *S. ternatum*. Finally, molecular docking was used to validate the interactions between the active compounds and the target. The results of our study lay a solid theoretical foundation for further research on the pharmaceutical and cosmetic applications of *S. ternatum* and may also contribute to the rational utilization of different parts of medicinal plants.

## 2. Results and Discussion

### 2.1. Phytochemical Characterization

The yields of ethanol extracts from *S. ternatum* whole plants or specific organs after vacuum treatment are shown in Table 1. The *S. ternatum* stems had the highest yield of 39.79 ± 4.93%, while the roots had the lowest yield of 17.99 ± 0.22%. The Folin–Ciocalteu and AlCl_3_ methods were used to evaluate the total phenol and flavonoid contents in the different *S. ternatum* parts, respectively. Compared with the other plant parts, the stems were poor in phenols and flavonoids. The *S. ternatum* leaves are particularly rich in flavonoids. These results suggest that the different parts of *S. ternatum* vary considerably in their chemical composition.

### 2.2. Chromatographic Analyses

**Figure 1:**
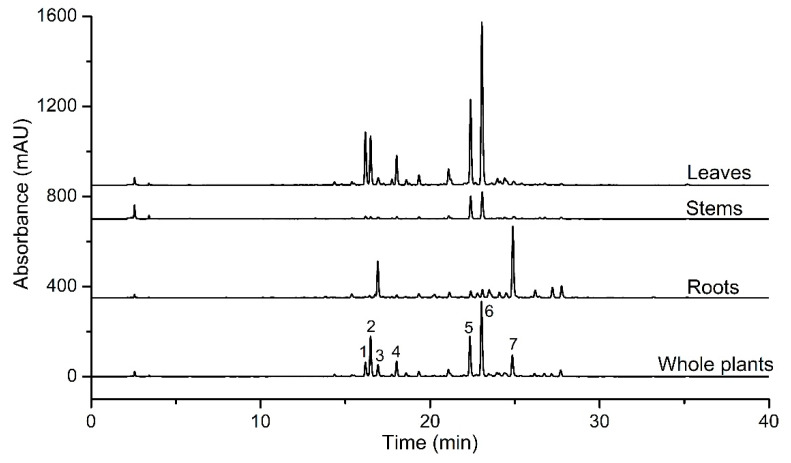
HPLC–UV chromatograms of ethanol extracts from whole plants, roots, stems, and leaves of *S. ternatum*.

Chromatographic analyses were performed to compare the distribution of secondary metabolites in different *S. ternatum* organs. As shown in Figure 1, the composition and abundance of the chromatographic peaks varied markedly in the chromatograms, indicating significant differences in secondary metabolites between the different *S. ternatum* organs. Generally, a relatively simple metabolic pattern was observed in the *S. ternatum* stem extract, whereas the leaf extract exhibited a more diverse profile. By comparing the retention time and mass spectra with those from our previous study [16], major common peaks were identified using HPLC coupled with electrospray ionization quadrupole time-of-flight mass spectrometry (HPLC–ESI-Q-TOF-MS/MS). As shown in Table 2, the peaks all represented flavonoid glycosides, with quercetin or kaempferol appearing as aglycones. These results indicate a specific distribution of secondary metabolites in different *S. ternatum* organs.

### 2.3. Antioxidant Activity

**Figure 2:**
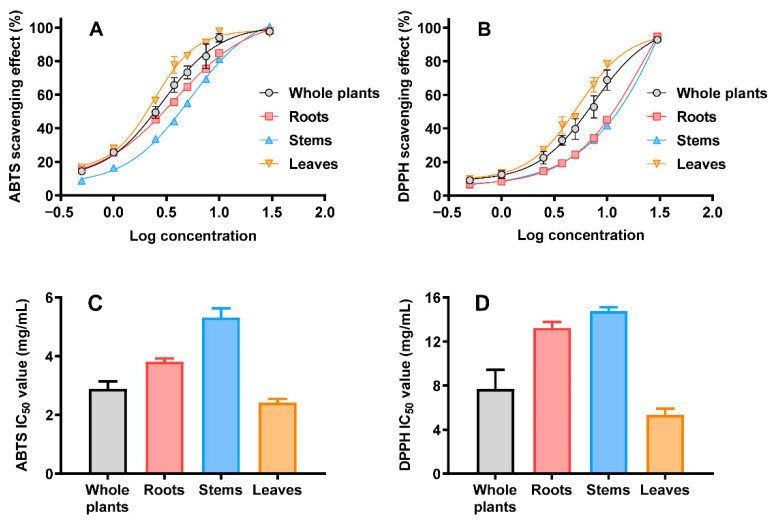
The radical scavenging ability of whole plants, roots, stems, and leaves from *S. ternatum*. ABTS (**A**) and DPPH (**B**) scavenging curves. The calculated IC_50_ values for ABTS (**C**) and DPPH (**D**). The data are shown as means ± SD (*n* = 3).

Figure 2A,B shows the 2,2ʹ-azino-bis (3-ethylbenzthiazoline-6-sulfonic acid) (ABTS) and 1,1-diphenyl-2-picrylhydrazyl (DPPH) radical scavenging abilities of the dried plant extracts, respectively. All ethanol extracts from different parts of *S. ternatum* exerted a radical-scavenging effect in a dose-dependent manner. The ABTS IC_50_ values of the ethanol extracts from whole plants, roots, stems, and leaves were 2.90 ± 0.25, 3.81 ± 0.11, 5.32 ± 0.31, and 2.43 ± 0.13 mg/mL, respectively (Figure 2C). The DPPH IC_50_ values of the ethanol extracts from whole plants, roots, stems, and leaves were 7.68 ± 1.76, 13.25 ± 0.54, 14.75 ± 0.36, and 5.36 ± 0.54 mg/mL, respectively (Figure 2D). Compared with L-ascorbic acid (VC) and 2,6-di-tert-butyl-4-methylphenol (BHT), the ABTS and DPPH IC_50_ values of the extracts were much higher. The ABTS IC_50_ values of VC and BHT were 75.44 ± 1.00 and 179.37 ± 5.92 μg/mL, respectively. The DPPH IC_50_ values of VC and BHT were 111.57 ± 2.25 and 223.80 ± 5.51 μg/mL, respectively. In summary, the ethanol extracts from different parts of *S. ternatum* presented a moderate radical scavenging ability. Meanwhile, the leaves showed higher antioxidant activity than the roots and stems. This may be related to the total phenol and flavonoid contents, which have significant antioxidant activities. As mentioned above, the *S. ternatum* leaves contained the highest phenol and flavonoid levels.

### 2.4. Anti-Inflammatory Activity

**Figure 3:**
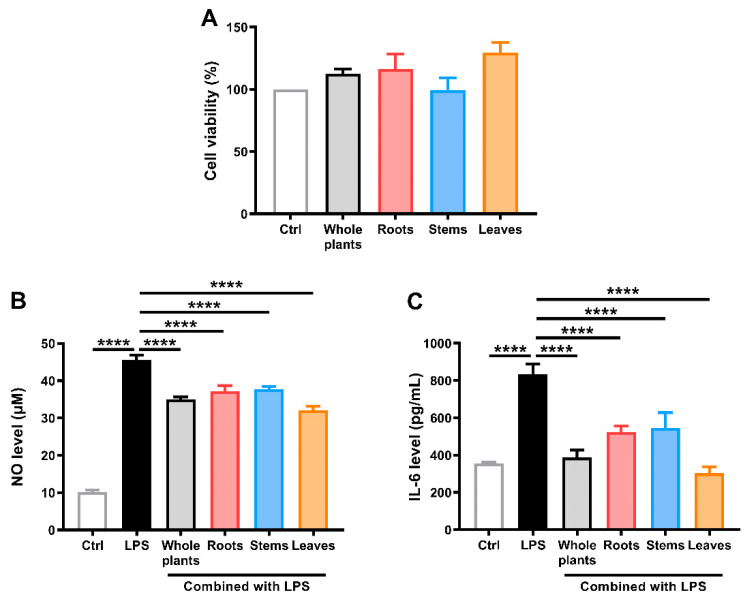
Assessment of the anti-inflammatory activities of the whole plants, roots, stems, and leaves from *S. ternatum*. (**A**) RAW 264.7 cell viability detected through the CCK-8 assay. (**B**) NO level measurement. (**C**) IL-6 level measurement. The data are shown as means ± SD (*n* = 3). **** *p* < 0.0001 vs. the LPS-stimulated group.

First, the cytotoxicity of the ethanol extracts from different parts of *S. ternatum* to RAW 264.7 cells was investigated using the Cell Counting Kit-8 (CCK-8) assay (Figure 3A). Cells were treated with the extracts (50 μg/mL) for 25 h. All plant extracts demonstrated no cytotoxicity to RAW 264.7 cells. Then, the anti-inflammatory properties of the plant extracts were studied in a model of lipopolysaccharide (LPS)-stimulated RAW 264.7 macrophages, and nitric oxide (NO) and interleukin-6 (IL-6) levels were evaluated. As shown in Figure 3B,C, NO and IL-6 levels increased significantly in the supernatant of LPS-stimulated cells (*p* < 0.0001). Co-treatment with ethanol extracts from different parts of *S. ternatum* decreased the inflammatory marker levels. Compared with the LPS-stimulated group, cells co-treated with ethanol extracts from the whole plants, roots, stems, and leaves of *S. ternatum* showed 29.81%, 23.59%, 22.07%, and 38.15% reductions in NO production, respectively. Similarly, cells co-treated with these ethanol extracts showed 93.18%, 64.93%, 60.26%, and 110.86% reductions in IL-6 release, respectively. These results suggest that the ethanol extracts from different parts of *S. ternatum* exerted obvious anti-inflammatory activity (*p* < 0.0001). Notably, the leaf extract exhibited the strongest effect.

### 2.5. Spectrum-Effect Relationship Analysis

**Figure 4:**
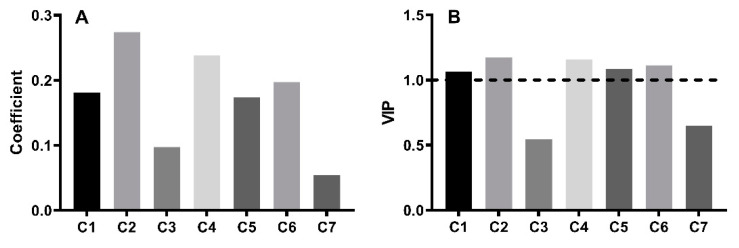
Spectrum-effect relationship analysis between the phytochemical profile and IL-6 level: (**A**) Regression coefficient. (**B**) VIP value.

To evaluate the correlation between the phytochemical profile and anti-inflammatory activity, spectrum-effect relationship analysis was performed using partial least squares regression (PLSR). IL-6 has been recognized as a key marker of inflammation [19,20]. Thus, its level was selected as the dependent variable for model development. The regression coefficient of the established PLSR model is displayed in Figure 4A. All seven compounds are positively correlated with an IL-6 reduction. C2 shows the highest coefficient (*R* = 0.274), followed by C4 (*R* = 0.239) and C6 (*R* = 0.197). Figure 4B demonstrates the variable importance in the projection (VIP) of each compound. The VIP values of C2, C4, C6, C5, and C1 are > 1, indicating that these compounds have a significant impact on the reduction in IL-6. Of which, C2 made the largest contribution to the model (VIP = 1.175). The obtained results suggest that C2 exhibited the strongest anti-inflammatory activity.

### 2.6. Molecular Modeling

**Figure 5:**
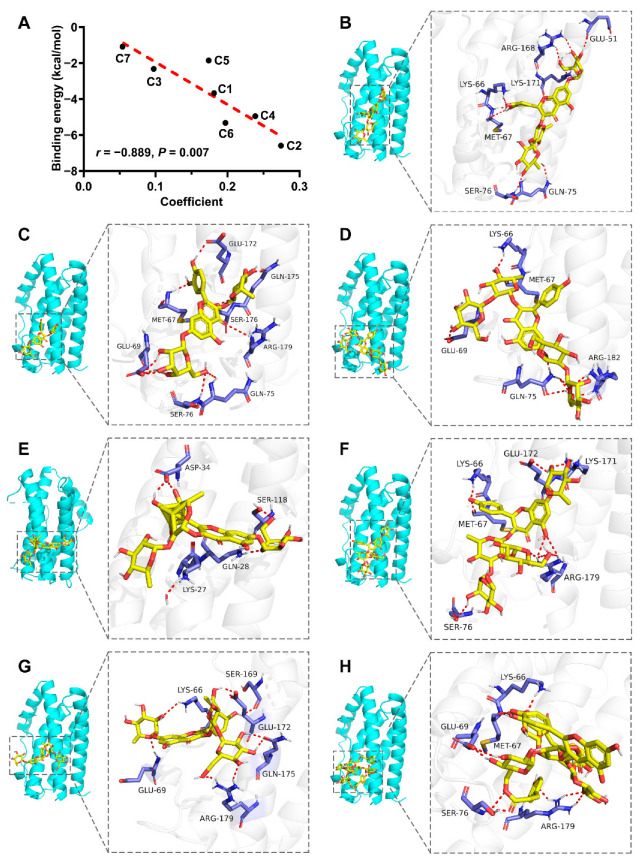
(**A**) Correlation analysis of the binding energy and the coefficient. (**B**–**H**) Docking patterns of the seven compounds and IL-6. (**B**) C1; (**C**) C2; (**D**) C3; (**E**) C4; (**F**) C5; (**G**) C6; (**H**) C7.

To further validate the anti-inflammatory activities of compounds C1−C7, molecular docking was employed to evaluate the interactions between the compounds and their anti-inflammatory target. Similarly, IL-6 was selected as a representative anti-inflammatory target for molecular docking in this study because of its importance in inflammation. Site I in IL-6 is the binding domain of IL-6R and is closely associated with classic signaling pathways [21,22]. Therefore, a grid box (126 Å × 126 Å × 114 Å, 0.375 Å spacing) was constructed around site I and centered at the following coordinates: x = 1.301, y = −19.932, z = 8.838. The docking results of the seven compounds with IL-6 (PDB ID:1ALU) are presented in Table 3. The free binding scores ranged from −1.10 to −6.60 kcal/mol. Additionally, Pearson’s correlation analysis was performed to assess the relationship between the binding energy and the coefficient obtained from the spectrum-effect relationship analysis [23]. As shown in Figure 5A, the binding energy was highly related to the coefficient (*r* = −0.889, *p* = 0.007). Thus, the molecular docking results were consistent with the conclusion of the spectrum-effect relationship analysis.

A lower binding energy indicates better binding affinity with the active site [24]. C2 exhibited the lowest binding energy, implying the most stable binding with IL-6. C2 also demonstrated the best anti-inflammatory activity. The 3D docking patterns of the seven compounds and IL-6 are shown in Figure 5B–H. As shown in Figure 5C, the high affinity between C2 and IL-6 was mainly attributed to hydrogen bonding with GLU-172, GLN-175, SER-176, ARG-179, GLN-75, SER-76, GLU-69, and MET-67.

## 3. Materials and Methods

### 3.1. Chemicals and Reagents

VC, Folin–Ciocalteu reagent, and dimethyl sulfoxide (DMSO) were purchased from Sigma–Aldrich (St. Louis, MO, USA). Sodium carbonate aqueous solution (Na_2_CO_3_, 1.0 M), aluminum chloride hexahydrate (AlCl_3_·6H_2_O), potassium acetate (CH_3_COOK), ABTS, potassium persulfate (K_2_S_2_O_8_), BHT, and GA were procured from Aladdin (Shanghai, China). DPPH was purchased from Yuanye (Shanghai, China). LPS was purchased from Beyotime (Shanghai, China). QE was purchased from Desite (Chengdu, China). Acetonitrile was of high-purity spectroscopic grade and was obtained from Merck (Darmstadt, Germany). Other chemicals and solvents were of analytical grade and were procured locally.

### 3.2. Plant Material and S. ternatum Extraction Protocol

Whole *S. ternatum* was collected during the winter of 2020 in Taishun County, Wenzhou, China. The collected plants were identified using pertinent literature and authenticated through comparison with the species preserved in our laboratory [16]. The whole plants or different parts of *S. ternatum* were ground and sieved to a fine powder after being completely air-dried. Then, 1 g of powder from each part was extracted twice using ultrasound with 10 mL of 70% aqueous ethanol for 30 min each. The supernatants were combined, filtered, and vacuum-dried at a low temperature. Three replicates were performed for each experiment. The residue powders of each part were stored at −30 °C until further use.

### 3.3. Spectrophotometric Analysis

All determinations were performed in 96-well plates using a BioTek EPOCH 2 microplate reader.

#### 3.3.1. Determination of Total Phenols

The total phenolic content of ethanol extracts from different parts of *S. ternatum* was determined using a modified Folin–Ciocalteu method [25]. Briefly, 75 µL of Folin–Ciocalteu reagent (15%, diluted with ultrapure water) was added to 40 µL of a suitable dissolved extract (6 mg/mL, dissolved in 30% methanol). After proper mixing, 85 µL of a Na_2_CO_3_ aqueous solution (1.0 M) was added to the mixture. The 96-well plate containing the mixture was further incubated at 45 °C for 15 min, and the absorbance at 765 nm was recorded. The total phenolic content was expressed as GA equivalents (µg GA equivalents per mg of dry extract) through a calibration curve, with GA ranging from 10 to 250 µg/mL (R^2^ = 1.00).

#### 3.3.2. Determination of Total Flavonoids

The total flavonoid content of the extracts was determined through the AlCl_3_ method, using QE as a standard [25]. Briefly, 60 µL of ethanol (95%), 40 μL of AlCl_3_ (1%), 40 μL of CH_3_COOK (0.1 M), and 60 μL of pure water were successively added to 40 µL of dissolved extract (6 mg/mL, dissolved in 30% methanol). The 96-well plate containing the mixture was incubated at room temperature for 30 min, and the absorbance at 415 nm was recorded. The total flavonoid content was expressed as QE equivalents (µg QE equivalents per mg of dry extract) through a calibration curve, with QE ranging from 10 to 500 µg/mL (R^2^ = 1.00).

#### 3.3.3. ABTS Radical Scavenging Assay

The ABTS radical scavenging assay was performed as described in our previous study [16]. Briefly, equal volumes of ABTS aqueous solution (6.83 mM) and K_2_S_2_O_8_ aqueous solution (2.47 mM) were mixed and incubated in the dark for 16 h to prepare ABTS radical stock solutions. The ABTS radical working solution was prepared via 25-fold dilution of the stock solution with ultrapure water. In the assay, the dried plant extract was diluted in 30% methanol at eight concentrations ranging from 0.5 to 30 mg/mL, and 10 μL of each sample was then added to 150 μL of ABTS radical working solution. The mixture was incubated at room temperature for 30 min before measuring the absorbance at 734 nm. The ABTS scavenging activity was calculated as (A_control_ − A_sample_)/A_control_ × 100%, where A_control_ is the absorbance of the control, and A_sample_ is the absorbance of the sample.

#### 3.3.4. DPPH Radical Scavenging Assay

The DPPH radical scavenging assay was performed as described in our previous study [26]. Briefly, the dried plant extract was diluted in 30% methanol at eight concentrations ranging from 0.5 to 30 mg/mL, and 10 μL of each sample was then added to 150 μL of DPPH methanol solution (150 µM). The mixture was incubated for 45 min in the dark before measuring the absorbance at 517 nm. The DPPH scavenging activity was calculated as (A_control_ − A_sample_)/A_control_ × 100%, where A_control_ is the absorbance of the control, and A_sample_ is the absorbance of the sample.

### 3.4. HPLC–UV and HPLC–ESI-Q-TOF-MS/MS Analyses

Dried ethanol extracts from different parts of *S. ternatum* were dissolved in 30% methanol to a final concentration of 15 mg/mL. High-performance liquid chromatography–ultraviolet (HPLC–UV) and HPLC–ESI-Q-TOF-MS/MS analyses were carried out using the same chromatographic and mass spectrometric conditions as those described in our previous work [16]. Briefly, HPLC–UV analysis was carried out using an Agilent 1260 HPLC system. Chromatographic separations were performed on a Waters Xselect HSS T3 column (4.6 mm × 150 mm, 3.5 μm) using a gradient mobile phase consisting of 0.1% formic acid in water (A) and acetonitrile (B) at a flow rate of 0.8 mL/min. The gradient elution program was performed as follows: 0–40 min, 5–35% B. The column was maintained at 35 °C, and the injection volume was 10 μL. The detection wavelength was set at 345 nm.

HPLC–ESI-Q-TOF-MS/MS analysis was carried out using an AB SCIEX TripleTOF 5600^+^ mass spectrometer equipped with a Waters Acquity UPLC system. Chromatographic conditions were the same with HPLC–UV. The experimental parameters of the Q-TOF-MS analysis were: scan mode, full scan; scan range, 100–2000 Da and 50–2000 Da for MS^1^ and MS^2^, respectively; ion spray voltage, +5500 V and −4500 V for positive and negative ion mode, respectively; ion source heater, 600 °C and 550 °C for positive and negative ion mode, respectively; collision energy, +40 V and −40 V for positive and negative ion mode, respectively; collision energy spread, 20 V; GS1, 50 psi; GS2, 50 psi; curtain gas, 30 psi. Data were processed using the Peakview software (AB SCIEX, version 1.2) provided with the instrument.

### 3.5. Cell Culture and Cell Viability Assay

Murine macrophage cells (RAW 264.7) were purchased from Meisen Chinese Tissue Culture Collections (MeisenCTCC, Hangzhou, China). The cells were cultured in Dulbecco’s modified Eagle’s medium supplemented with 10% fetal bovine serum (FBS) and penicillin–streptomycin (100 U/mL) in a humidified atmosphere (5% CO_2_, 37 °C). Dried ethanol extracts from different parts of *S. ternatum* were dissolved to 50 mg/mL in DMSO and diluted in culture medium before use. The cytotoxicity of *S. ternatum* to RAW 264.7 cells was evaluated using a CCK-8 assay. Briefly, 1 × 10^4^ cells/well were seeded in 96-well plates and incubated overnight. The medium was then replaced with FBS-free medium containing 50 μg/mL of extracts from different parts of *S. ternatum*. Cells treated with DMSO (0.1%) were used as controls. After 25 h of treatment, 10 μL of CCK-8 solution (GLPBIO, Montclair, NJ, USA) was added to each well and incubated for 4 h. The optical density (OD) was measured at 450 nm. Cell viability (%) = OD_sample_/OD_control_ × 100, where OD_sample_ is the OD value of the treated sample, and OD_control_ is the OD value of the control.

### 3.6. NO Assay and Enzyme-Linked Immunosorbent Assay (ELISA)

RAW 264.7 cells (5 × 10^5^ cells/well) were grown in a 12-well plate overnight. Thereafter, the medium was replaced with FBS-free medium containing 50 μg/mL of extracts from different parts of S. ternatum. After 1 h of incubation, the cells were stimulated with LPS (1 μg/mL) for 24 h. NO level in the supernatant was measured by the Griess method using Griess reagent (Beyotime, Shanghai, China). Briefly, 50 µL of Griess Reagent I and 50 μL of Griess Reagent II were successively added to 50 µL of supernatant, and the absorbance at 540 nm was recorded. The NO concentration was calculated through a calibration curve, with sodium nitrite ranging from 1 to 100 µM (R^2^ = 1.00). IL-6 level in the supernatant was measured using a mouse IL-6 ELISA Kit (Beyotime, Shanghai, China) according to the manufacturer’s protocol. 

### 3.7. Spectrum-Effect Relationship Analysis

The spectrum-effect relationship between the phytochemical profiles and biological activities of the ethanol extracts from different parts of *S. ternatum* was analyzed using PLSR. The common chromatographic peak areas were defined as independent variables (*X*), while the reduction in the IL-6 level was set as a dependent variable (*Y*). SIMCA 14.1 software was used to establish the PLSR model. The regression coefficient was regarded as an index that exhibited the relative impact of the independent variables on the dependent variables. The VIP value was used to identify chromatographic peaks significantly correlated with the biological activities of the extracts.

### 3.8. Compound-Target Molecular Modeling

Molecular docking was performed to compare the anti-inflammatory activities of the major phytochemicals in *S. ternatum*. Briefly, the docking steps were as follows. (1) The compound structure (sdf format) was obtained from the PubChem database (https://pubchem.ncbi.nlm.nih.gov/ (accessed on 3 May 2022).). The 3D conformation was constructed using ChemBio3D Ultra 12.0 software (CambridgeSoft Corp., Waltham, MA, USA), and the energy was minimized using MM2 force field optimization [27]. Then, the 3D structure was hydrogenated and saved in pdbqt file format using AutoDockTools 1.5.6 (http://mgltools.scripps.edu/ (accessed on 2 August 2021)). (2) The crystal structure of the anti-inflammatory target was downloaded from the RCSB PDB database (https://www.rcsb.org/ (accessed on 9 May 2022)) and imported into PyMOL 2.5.0 software (Schrödinger, LLC, New York, NY, USA) to remove the solvent molecules and ligands. The receptor structure was further modified by deleting water molecules and adding all polar hydrogens using AutoDockTools, and a docking grid box was subsequently constructed at the active site. (3) AutoDockTools was employed to investigate the molecular interactions between compounds and targets, as well as to calculate the binding energy. The best conformer was obtained using the Lamarckian genetic algorithm in flexible docking [19,28]. (4) The conformation with the lowest binding energy was considered the most suitable binding mode and was visualized using PyMOL.

### 3.9. Statistical Analysis

Statistical analysis was performed using GraphPad Prism software (version 6.0; La Jolla, CA, USA). The data are expressed as means ± SD. The level of significance between groups was determined using one-way analysis of variance, followed by Fisher’s LSD multiple comparison. Results with *p* < 0.05 were considered statistically significant.

## 4. Conclusions

In this study, a spectrum-effect relationship-based strategy was used to explore the bioactive basis and tissue distribution in *S. ternatum*. The chemical profiles and the antioxidant and anti-inflammatory properties of ethanol extracts from roots, stems, and leaves of *S. ternatum* were comparatively analyzed. The leaves contained the most abundant flavonoids and exerted the best biological activities. Spectrum-effect relationship analysis and molecular docking indicated that quercetin 3-*O*-rhamnoside-7-*O*-glucoside (C2) possess high anti-inflammatory activity by binding with IL-6. These results show that *S. ternatum* is a rich source of bioactive flavonoids with potential exploitation in pharmaceutical and cosmetic industries.

## Figures and Tables

**Table 1:** Extraction yields, total phenols, and total flavonoids of ethanol extracts from *S. ternatum* whole plants or specific organs.

Plant Part	Yield	Total Phenols	Total Flavonoids
	%	μg GA/mg Dry Extract	μg QE/mg Dry Extract
Whole plants	21.39 ± 1.05	20.32 ± 1.27	7.96 ± 0.87
Roots	17.99 ± 0.22	20.80 ± 0.35 ^b^	3.29 ± 0.19 ^d^
Stems	39.79 ± 4.93	14.49 ± 0.34	2.51 ± 0.09
Leaves	26.19 ± 2.39	22.24 ± 0.82 ^a,b^	12.87 ± 0.40 ^b,c^

GA: gallic acid; QE: quercetin. Data are shown as the means ± SD (*n* = 3). ^a^ *p* < 0.05 vs. roots; ^b^ *p* < 0.0001 vs. stems; ^c^ *p* < 0.0001 vs. roots; ^d^ *p* < 0.05 vs. stems.

**Table 2:** Identification of the main phytochemicals in *S. ternatum* using HPLC–ESI-Q-TOF-MS/MS.

No.	*t*_R_ (min)	Identification	Formula	Selected Ion	Measured *m/z*	Error (ppm)	MS^2^
C1	16.188	Quercetin 3-*O*-glucosyl-(1→2)-rhamnoside-7-*O*-glucoside	C_33_H_40_O_21_	[M + H]^+^	773.2142	0.9	611.1621, 465.1041, 303.0500
				[M − H]^−^	771.1994	0.6	609.1544, 462.0839, 301.0360, 300.0283, 299.0198
C2	16.489	Quercetin 3-*O*-rhamnoside-7-*O*-glucoside	C_27_H_30_O_16_	[M + H]^+^	611.1606	−0.1	465.1004, 303.0486
				[M − H]^−^	609.1461	−0.7	463.0943, 462.0843, 447.0981, 301.0364, 300.0283, 299.0203
C3	16.925	Kaempferol 3-*O*-glucosyl-(1→2)-rhamnoside-7-*O*-glucosyl-(1→2)-glucoside	C_39_H_50_O_25_	[M + H]^+^	919.2735	2.3	757.2162, 611.1598, 449.1068, 433.1130, 287.0541
				[M − H]^−^	917.2578	1.0	593.1622
C4	18.026	Kaempferol 3-*O*-glucosyl-(1→2)-rhamnoside-7-*O*-glucoside	C_33_H_40_O_20_	[M + H]^+^	757.2192	0.8	595.1667, 449.1080, 287.0545
				[M − H]^−^	755.2044	0.5	593.1579, 446.0886, 285.0401, 284.0327, 283.0251
C5	22.354	Kaempferol 3-*O*-(2,3-di-*O*-glucosyl)-rhamnoside-7-*O*-rhamnoside	C_39_H_50_O_24_	[M + H]^+^	903.2776	1.2	741.2276, 595.1672, 433.1132, 287.0551
				[M − H]^−^	901.2629	1.1	755.2154, 284.0326
C6	23.036	Kaempferol 3-*O*-glucosyl-(1→2)-rhamnoside-7-*O*-rhamnoside	C_33_H_40_O_19_	[M + H]^+^	741.2251	1.9	433.1141, 287.0552
				[M − H]^−^	739.2095	0.5	593.1583, 430.0932, 413.0913, 285.0401, 284.0327, 283.0248
C7	24.855	Kaempferol 3-*O*-[glucosyl-(1→4)]-[6-*O*-[4-hydroxy-(*E*)-cinnamoyl]-glucosyl-(1→3)]-6-*O*-[4-hydroxy-(*E*)-cinnamoyl]-glucosyl-(1→2)-rhamnoside	C_57_H_62_O_29_	[M + H]^+^	1211.3524	6.1	903.2617, 763.2479, 595.1625, 455.1514, 449.1094, 419.1353, 309.0975, 287.0550
				[M − H]^−^	1209.3324	1.7	1047.3040, 901.2650

**Table 3:** Docking results between the main compounds in *S. ternatum* and IL-6.

No.	PubChem CID	Binding Energy (kcal/mol)
C1	74978226	−3.68
C2	14484601	−6.60
C3	78100944	−2.33
C4	74978107	−4.96
C5	78100943	−1.86
C6	162950015	−5.33
C7	78100892	−1.10

## Data Availability

The data presented in this study are available on request from the corresponding author. The data are not publicly available due to privacy.

## Sample Availability

Samples of the compounds are available from the authors.
